# Concurrent follicular lymphoma and Langerhans cell sarcoma

**DOI:** 10.1002/jha2.287

**Published:** 2021-09-09

**Authors:** Mehrnoosh Tashakori, L. Jeffrey Medeiros

**Affiliations:** ^1^ Department of Hematopathology The University of Texas MD Anderson Cancer Center Houston Texas USA

**FIGURE 1:**
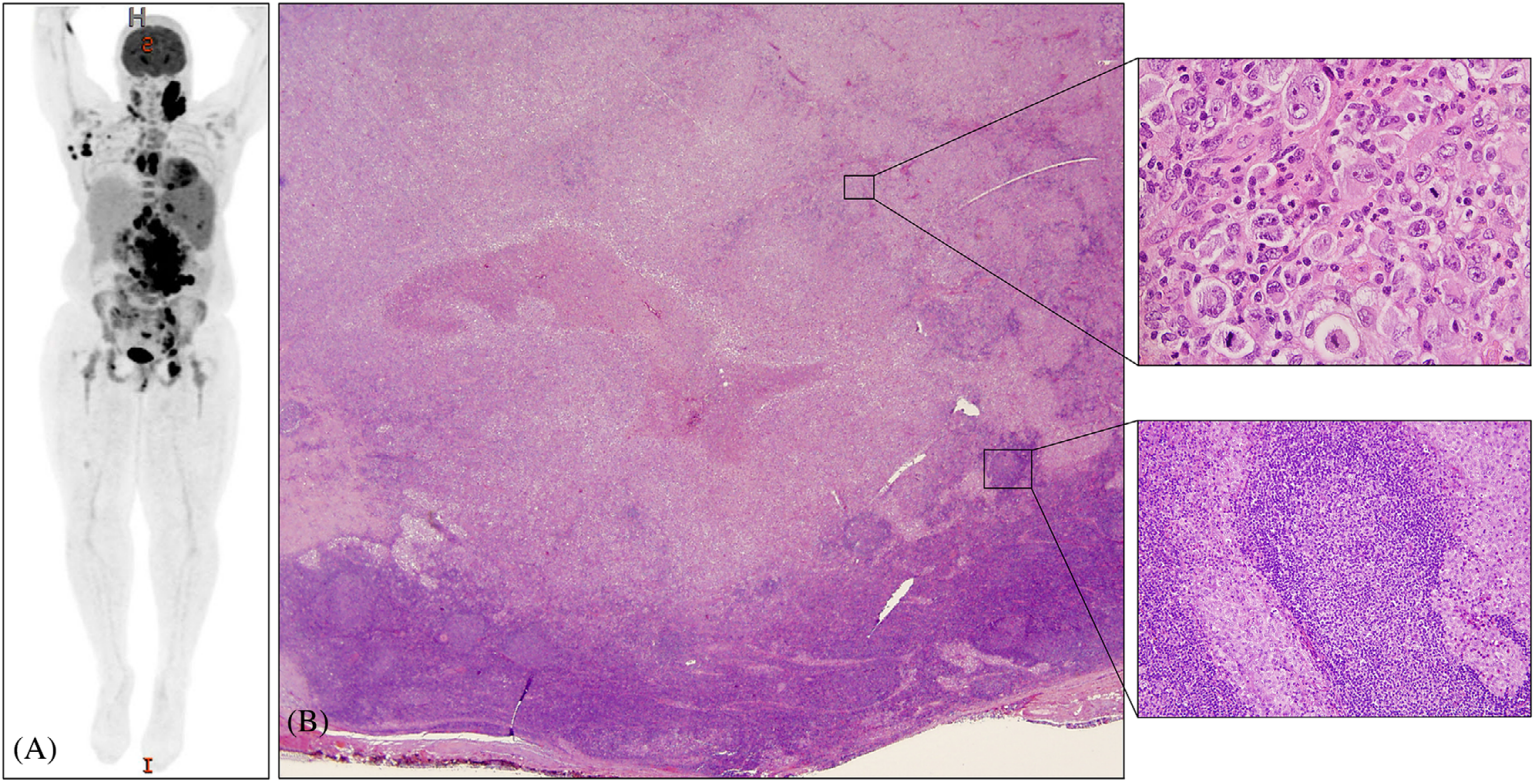
PET/CT scan (left) shows discordant SUV uptake above and below diaphragm. Lymph node biopsy (middle image) shows Langerhans cell sarcoma (upper part of field) and follicular lymphoma (lower part of field). High magnification shows Langerhans cell sarcoma (upper right image) and follicular lymphoma surrounded by Langerhans cells (lower right image).

A 31‐year‐old woman presented with abdominal pain and a recent history of chest pain and B symptoms. Physical examination showed lymphadenopathy on left side of face/upper neck and mild splenomegaly. Laboratory workup showed microcytic hypochromic anemia (hemoglobin 9.8 mg/dl, and MCV: 72 fl), and elevated serum lactate dehydrogenase of 366 U/L. Positron emission tomography/computed tomography (PET/CT) imaging showed dichotomous ^18^F‐fluorodeoxyglucose avidity (Figure 1, Panel A). The standardized uptake value (SUV) was 20.9 above the diaphragm, and about 7 below the diaphragm; a splenic lesion had an SUV of 5.2. The dichotomous SUV suggested different pathologies above and below diaphragm. The patient underwent biopsy of axillary and retroperitoneal lymph nodes. Histology and extensive immunophenotypic work‐up revealed concurrent follicular lymphoma and Langerhans cell sarcoma in both specimens (Figure 1, Panel B). Both tumors carried *IGH/BCL2*, suggestive of a clonal relationship. The patient was treated initially with six cycles of the R‐CHOP (rituximab, cyclophosphamide, doxorubicin, vincristine, and prednisone) regimen, which resulted in remission of the follicular lymphoma (FL). She is currently receiving the ICE (ifosfamide, carboplatin, and etoposide) regimen for persistent Langerhans cell sarcoma (LCS). This case highlights the phenomenon of transdifferentiation, in which neoplastic B cells are thought to convert to a histiocytic or Langerhans cell phenotype via unknown mechanisms. The case also highlights the value of histopathologic examination of multiple biopsy specimens for accurate diagnosis and design of a treatment plan in patients in whom PET/CT shows dichotomous SUV values.

